# Ontology-based time information representation of vaccine adverse events in VAERS for temporal analysis

**DOI:** 10.1186/2041-1480-3-13

**Published:** 2012-12-20

**Authors:** Cui Tao, Yongqun He, Hannah Yang, Gregory A Poland, Christopher G Chute

**Affiliations:** 1 Division of Biomedical Statistics and Informatics, Department of Health Sciences Research, Mayo Clinic, Rochester, MN, USA; 2 Unit for Laboratory Animal Medicine, Department of Microbiology and Immunology, and Center for Computational Medicine and Bioinformatics, University of Michigan Medical School, Ann Arbor, MI, USA; 3 Mayo Vaccine Research Group, Mayo Clinic, Rochester, MN, USA

## Abstract

**Background:**

The U.S. FDA/CDC Vaccine Adverse Event Reporting System (VAERS) provides a valuable data source for post-vaccination adverse event analyses. The structured data in the system has been widely used, but the information in the write-up narratives is rarely included in these kinds of analyses. In fact, the unstructured nature of the narratives makes the data embedded in them difficult to be used for any further studies.

**Results:**

We developed an ontology-based approach to represent the data in the narratives in a “machine-understandable” way, so that it can be easily queried and further analyzed. Our focus is the time aspect in the data for time trending analysis. The Time Event Ontology (TEO), Ontology of Adverse Events (OAE), and Vaccine Ontology (VO) are leveraged for the semantic representation of this purpose. A VAERS case report is presented as a use case for the ontological representations. The advantages of using our ontology-based Semantic web representation and data analysis are emphasized.

**Conclusions:**

We believe that representing both the structured data and the data from write-up narratives in an integrated, unified, and “machine-understandable” way can improve research for vaccine safety analyses, causality assessments, and retrospective studies.

## Background

Effective analyses of time trends for post-vaccine adverse events (AEs) can enhance clinical research in different areas such as vaccine safety analyses, causality assessments, and retrospective studies. The FDA/CDC Vaccine Adverse Event Reporting System (VAERS) [1] provides a valuable data set for these purposes. VAERS maintains a database for reports of AEs following vaccination. These reports contain both structured data (e.g., gender, age, vaccination date, and onset date), as well as short narratives that usually provide more detailed descriptions of the vaccination, the related events, and their time constraints.

The structured data in the VAERS database have been widely leveraged in different medical analyses for vaccine adverse events [2,3]. The unstructured nature of the narratives, however, makes the data embedded in them difficult for use in further analyses. These narratives usually contain additional valuable information (e.g., patient ages that were not reported in a structured way, vaccination doses, and durations or time stamps for multiple events following vaccination) that could potentially lead to more effective and concrete clinical analyses, and perhaps important clinical insights.

It is challenging, however, to process this time-related data hidden in the narratives from the AE reports. The VAERS receives 30,000 reports annually. Manually processing these reports is tedious and expensive. Even if the related information has been successfully marked and extracted, the temporal relations needed for time pattern recognition are often not explicitly expressed in the original documents, but rather need to be inferred.

**Figure 1:**
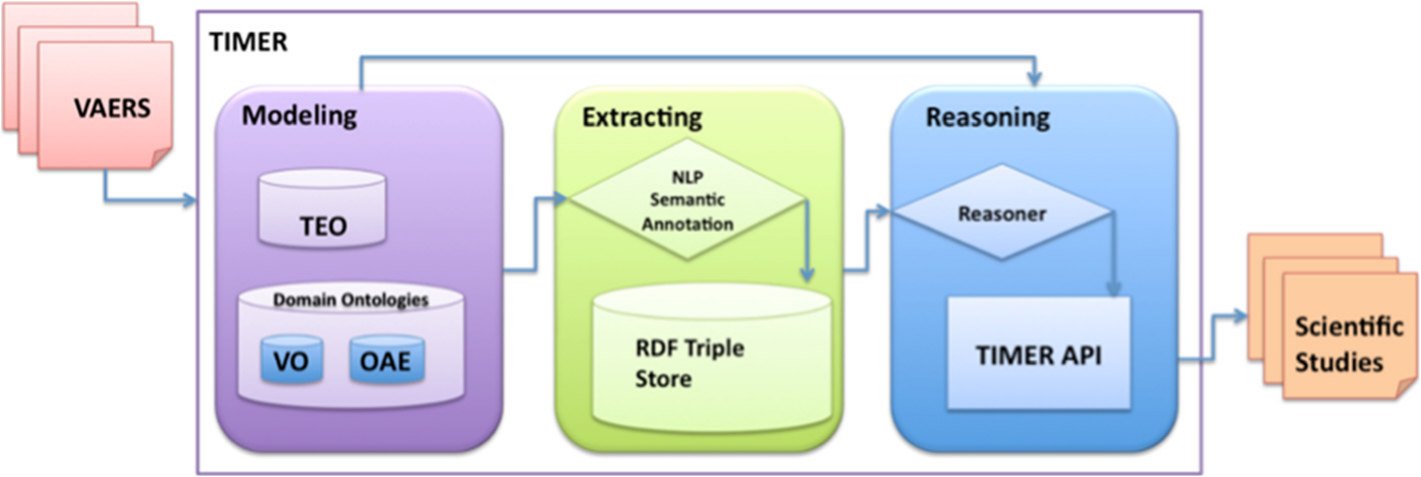
TIMER system overview.

Targeting these challenges, we have designed the Temporal Information Modeling, Extraction, and Reasoning (TIMER) framework for extracting, querying, and inferring useful temporal information automatically. Figure 1 shows the TIMER system overview. One core component of TIMER is the modeling component. Our vision is to leverage ontologies to semantically model the domain and time knowledge. TIMER relies on the ontologies as the annotation schema for its extraction component, and as the knowledge base for its reasoning component. It is essential to ensure that the ontologies are capable of representing related data faithfully in an integrated, machine-understandable way, so that computer programs can automatically process the data, infer new knowledge, sort clinically relevant events over the timeline, and facilitate data querying for clinical research analyses. In this paper, we introduce our efforts to leverage Semantic Web mechanisms to represent time-related information for vaccine adverse events from the VAERS database. We use the Ontology of Adverse Events (OAE, previously named Ontology of Adverse Event or AEO) [4] and the Vaccine Ontology (VO) [5] for representing vaccine names and event names in a standard way. We use the Time Event Ontology (TEO) [6] to represent the time information among the events.

Previous research indicated that Semantic Web tools and technologies provide a viable solution for modeling of heterogeneous data, conducting scalable querying over the data, and inferring new knowledge [7-10]. We believe there are some unique benefits to applying these semantic web techniques to healthcare data: (1) the World Wide Web Consortium (W3C) recommendations provide a shared set of constructs which enable better interoperability between applications that exchange machine-understandable information; (2) the Web Ontology Language (OWL)’s formal semantics offer consistency checking ability for data represented using it; (3) the decidability and computability features of OWL-DL (Description Logic) can provide enough expressiveness for semantically defining concepts and their relationships that can support reasoning; (4) the Rule Interchange Format (RIF) provides a concrete expression language for rules defining clinical guidelines, as well as a standard for the interchanges between rules specified in different rule languages; and (5) the linked data feature of RDF graph can bring information concerning a particular instance together from heterogeneous sources [11].

The rest of the paper is organized as follows. We first introduce the three ontologies (TEO, OAE, and VO) and how they can be used to represent time in the area of vaccine adverse events. A VAERS case report is then presented as a use case for the ontological representations. The advantages of using our ontology-based Semantic web representation and data analysis are emphasized. Finally we draw conclusions and discuss further improvements.

## Ontology representation

### TEO and time relation reasoning^a^

**Figure 2:**
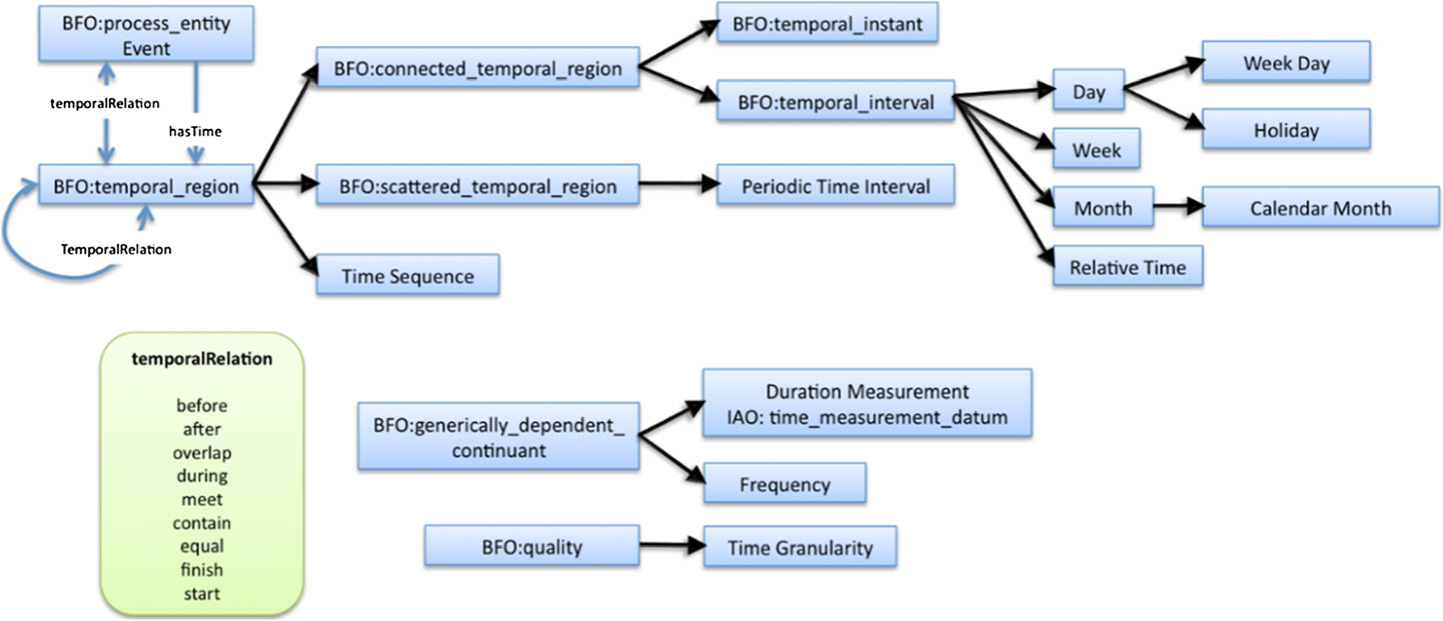
**High-level overview of the time event ontology, with high-level classes and their hierarchical information shown. **Please note that this overview does not show the low-level classes, the object properties other than temporal relations, or data type properties defined in the TEO.

The TEO is an OWL ontology designed for representing the temporal relations among events and time stamps. The TEO is an extended version of the Clinical Narrative Temporal Relation Ontology (CNTRO) [12,13], which is our effort representing basic temporal relations among events extracted from clinical narratives. We expanded the CNTRO to TEO since we believe that the time modeling should be general for all the domains. The TEO aims to model time event relationships in general, not specific to the clinical or biomedical domain. Figure 2 shows a high-level overview of the TEO. All the TEO concepts were derived from the Basic Formal Ontology (BFO) [14] to ensure upper-level interoperability with other ontologies. A TEO *event* is equivalent to the BFO term ‘processual_entity’ which is defined as: “an occurrent that exists in time by occurring or happening, has temporal parts and always involves and depends on some entity”. Events could be administration of medication (e.g., a vaccine), follow-up lab testing, patient complaints, adverse events with different symptom outcomes, etc. The temporal relations adopted from Allen's interval algebra [15,16] are defined using OWL object properties. Time stamps of when these events occurred can be represented if known via the *hasTime* relation. Duration information of a particular event (if it spanned minutes, hours, days) or between events can be annotated, as well as the temporal relationships between events implied throughout the narrative through the use of words such as “before”, “after”, “during”, etc. Granularity of a specific time stamp or frequency of the re-occurred time can also be modeled. In addition, the TEO also covers a periodic time interval which models the time of the events that repeat themselves (e.g., “Exercise 20 minutes 3 times/day starting from July 21 for 2 weeks”). It semantically defines specific temporal regions such as holidays, weekdays, etc, as well as specific temporal intervals such as days, weeks, months, today, tomorrow, etc. Here we use a few examples to illustrate how we can represent time-related information with respect to the TEO.

#### Time instants and intervals

With respect to TEO, we can assign any event a time stamp if available. We can represent “vaccinated w/MMR on July 6th” using the following triples:

1. <event1> rdf:type Event;

2. <event1> rdfs:label "vaccinated w/MMR ";

3. <event1> hasTime <tInst1>;

4. <tInst1> rdf:type TimeInstant;

5. <event1>hasOrigTime "July 6^th^";

6. <event1>hasNormalizedTime 2006-09-06;

7. <event1>hasGranularity "day";

Here we created a new OWL individual <event1>, which has type as *TEO*:*Event*, and label "vaccinated w/MMR". We further specify it has a timestamp <tInst1>, which has type *TEO*:*TimeInstant*. We can keep track of the original expression of this time instant using the property *hasOrigTime* and have the system normalize the time expression to a standard way and store it using *hasNormalizedTime*. The system can also infer the level of granularity of the time instant. In this example, it is “day”. Note that in this original text write-up, we do not know the year of the event (we only know July 6^th^), but the year can usually be inferred from the structured information from the same record.

Similarly, we can represent an event associated with a time interval. A time interval could have a start time, an end time, and a duration. Both start time and end time should be represented as an OWL individual of a time instants. For duration, we can specify the value and its unit (such as day, month, and year). For example, TEO represents “eighteen days” using the following triples:

1. <durat1> rdf:type Duration;

2. <durat1>hasValue "18";

3. <durat1>hasUnit "day";

#### Temporal relations

TEO allows users to represent temporal relations between two events. TEO adopts and adapts Allen's Interval Algebra [15,16] for representing temporal relations. Allen’s interval algebra defines temporal relations such as before, after, equal, overlap, meet, during, start, and finish between two time intervals. TEO, on the other hand, uses these relations between two events. For example, we can semantically represent “BRIEF GENERALIZED SEIZURE AFTER DTP” using the following triples:

1. <event1> rdf:type Event;

2. <event1> rdfs:label "BRIEF GENERALIZED SEIZURE";

3. <event2> rdf:type Event;

4. <event2> rdfs:label "DTP";

5. <event1> after <event2> ;

In many situations, we also want to add a time offset of this kind of temporal relations to describe the duration between the two events. For example, we can semantically express “Eighteen days after vaccination she developed a fever” using the following triples:

1. <event1> rdf:type Event;

2. <event1> rdfs:label "vaccination";

3. <event2> rdf:type Event;

4. <event2> rdfs:label "she developed a fever";

5. <event2> after <event1>

6. <a1> rdf:type owl:Axiom

7. <a1> owl:annotatedSource <event1>

8. <a1> owl:annotatedProperty after

9. <a1> owl:annotatedTarget <event2>

10. <a1> hasTimeOffset <durat1>

The TEO provides a representation mechanism to model temporal information stated within adverse event reports in a “machine-understandable” way. Sorting out the events on a timeline or answering time-related clinically significant question, however, usually cannot be accomplished by querying the information explicitly stated within the reports. Many times it requires semantic inference to fully answer the time-relevant questions.

The following is an example of narrative text within an adverse event report, which would require semantic reasoning:

“18 month-old vaccinated w/MMR on July 6^th^. Eighteen days after vaccination she developed a fever of 104 and macular rash of the face, torso & legs. Dx: vasculitis 2 wks later. Patient hospitalized 08-08”.

There are five events within this example: vaccinated w/MMR (event1), fever (event2), macular rash (event3), vasculitis (event4), and patient hospitalization (event5). The durations between the event1 and event2/event3 are explicitly stated, as well as the durations between event2/event3 and event4. The reader, however, needs to infer that patient hospitalization occurred after the vasculitis was diagnosed, which was after the vaccination, the fever, and the rash. A reader can also infer from the text for the actual dates of the fever, the rash, and the vasculitis diagnosis.

The TIMER system provides an Application Programming Interface (API) to infer this kind of temporal relation automatically after the information is represented in the Semantic Web notations.

### OAE and VO usage

The OAE is an OWL ontology for representing adverse events. The OAE has been developed by following the OBO Foundry principles including openness, collaboration, and use of a common shared syntax [17]. OAE is aligned with the top ontologies such as Basic Formal Ontology (BFO) [14] and the Relation Ontology (RO) [18].

In the current version of OAE, the term ‘adverse event’ is defined as a *pathological bodily process* (OGMS:0000061) that occurs after a medical intervention and is likely induced by the medical intervention. Examples of medical interventions include vaccination, drug administration, usage of medical devices, and surgery. An adverse event may or may not be caused by a medical intervention. In OAE, we specifically define a term ‘causal adverse event’ as a *pathological bodily process* that is induced/caused by a medical intervention. Currently, OAE has 2,464 representational units, annotated by means of 981 terms with specific such as OAE identifiers, and the other terms imported from existing ontologies including BFO, RO, and the Ontology of Biomedical Investigations (OBI) [19]. Importing external ontology terms ensures the semantic interoperability among ontologies and avoid duplicated terms to be generated.

The VO is a community-based ontology in the domain of vaccine and vaccination [20]. Like TEO and OAE, VO is developed in OWL and aligned with BFO and RO. VO has classified all existing vaccines licensed for human and animal uses in the U.S.A. and Canada. For each licensed vaccine, VO also includes relevant attributes such as vaccine type, vaccine component (e.g., antigen, adjuvant, and preservative), vaccination route, manufacturer, and the disease and pathogen targeted by the vaccine. All these data are organized in ontological format and shared syntax, supporting automated reasoning and SPARQL query.

MedDRA (http://www.meddramsso.com/) is used as the default controlled vocabulary for describing adverse event terms in VAERS. To better represent adverse events in OAE, we have made a match (cross-reference) between many OAE terms and MedDRA terms. Compared to MedDRA, OAE uses a formal ontology format with machine-parseable logical definitions and structures. OAE also imports vaccine-specific information from VO, making it an ideal platform for analyzing vaccine time events.

In order to make OAE, VO, and TEO work seamlessly, alignments between these three ontologies have been made. All these ontologies use BFO as the top ontology. TEO is imported to OAE as a middle-layer ontology for representation of time. All adverse events in OAE are subclasses of TEO events (*i*.*e*., BFO term ‘processual_entity’). Therefore, these adverse event classes in OAE automatically inherit TEO methods for time representation.

## Case evaluation

**Figure 3:**
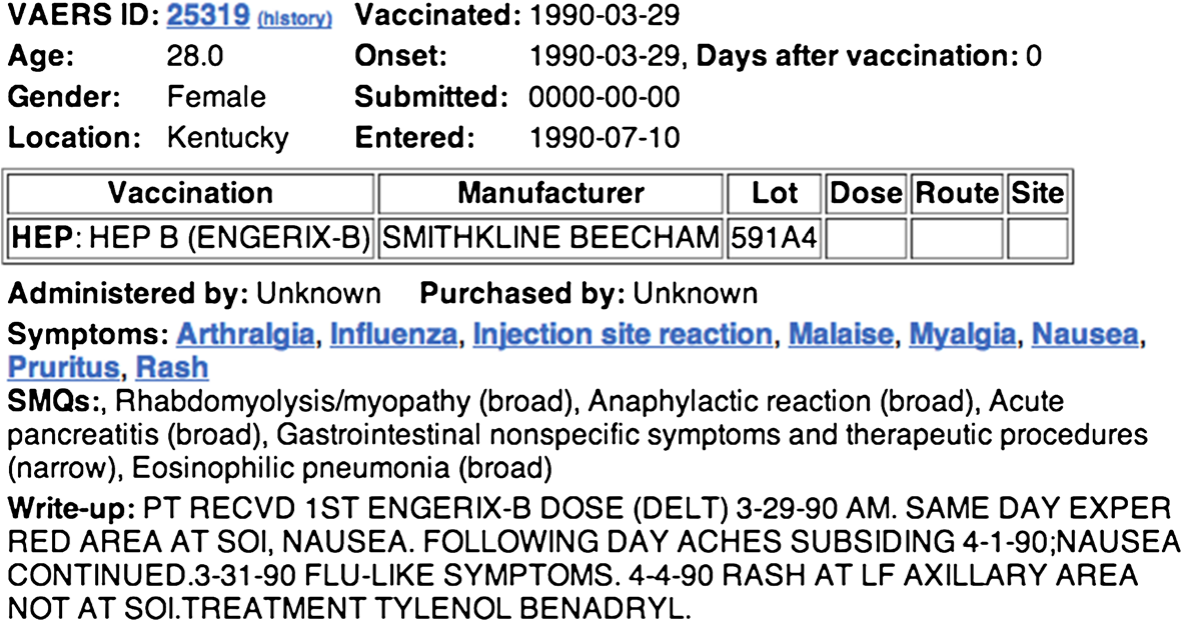
**A sample report from the VAERS database. **The report is semi-structured. Data such as patient's age, gender, location, the vaccination date, as well as some basic classifications is presented in structured way. But more detailed temporal information can only be retrieved from the write-up narratives. Original source: http://www.medalerts.org/vaersdb/findfield.php?IDNUMBER=25319.

Let us use the sample file in Figure 3 as a running use case to illustrate how we can use an ontology-based approach to represent and infer useful temporal information, as well as facilitate time trend analysis. The database provides dates such as “Vaccinated”. “Onset”, “Submitted”, and “Entered” in a structured way. However, these dates, in many cases, are not accurate or sufficient for time trend analysis. In the above example, we can observe that the onset date in the structured report is only one date: 1990-03-29. In the write-up, we can see there were actually a series of effects that happened after the vaccination, on different days. The additional information in the write-up narrative, however, is not easy to query or be processed by computers, or even by human experts. If the information of interest from the narratives is represented by the TEO ontology, it can be queried and processed easily for further analysis.

### OAE and VO modeling

OAE and VO can be used to represent adverse events and related vaccine information in an ontological format. The original case reports use MedDRA to represent symptoms. In OAE, an adverse event (AE) is a pathological bodily process that starts with a medical intervention (e.g., vaccination) and ends with the disscovery of a symptom (e.g., fever), a sign (e.g., increased blood cell count), or a process (e.g., influenza viral infection). The representation of an AE in OAE as such a whole process provides us ways to present various variables that contribute to the adverse events (e.g., patient age and sex, and vaccination dose and route) as well as the time intervals between different subprocesses. For example, we represent the nausea in Figure 3 as ‘nausea AE’ that occurs after a vaccination in a specific vaccine host. The OAE representation shows that the patient had adverse events in different areas (*e*.*g*., skin, joint, and digestive systems). It is noted that an ‘influenza AE’ may not be induced by influenza virus. The better term to represent the case is an OAE term ‘influenza like illness AE’ (OAE_0000100). The patient in the case reported was vaccinated with the vaccine Engerix-B, which is semantically defined in VO with a VO ID of VO_0010711. VO provides the hierarchical information as well as the semantic assertions associated for different vaccines. In the future, vaccine information can be directly imported to OAE to support efficient automated reasoning.

### TEO modeling of the time events in this case

**Figure 4:**
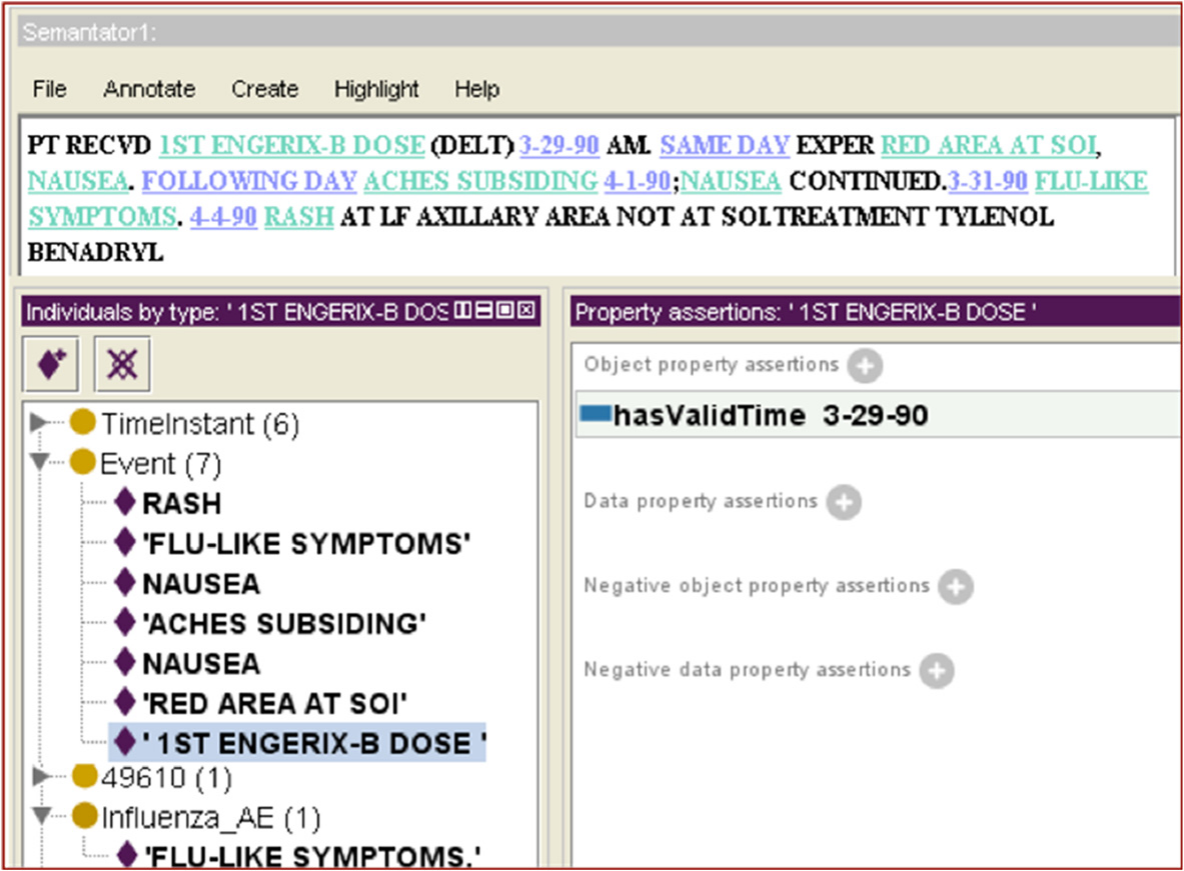
**The annotation results of the example in Figure **1 **(Partial): The upper panel shows the information of interest highlighted.** The bottom left panel shows classes with annotated instances. And the bottom right panel shows a recognized temporal relation between “1^st^ ENGERIX-B DOSE” and its time stamp. This figure is generated as a screenshot using the Semantator (http://informatics.mayo.edu/CNTRO/index.php/Semantator) semi-automatic annotation environment.

The recognized named entities will be annotated using terms in TEO as well as domain ontologies (OAE & VO) to create a set of attribute-value pairs. Figure 4 shows some examples. We have recognized 7 events (shown in the list) as well as 6 time instants (not shown) from the short narratives. These events are also annotated with respect to domain ontologies so that the computer program “understands” the semantic meaning of the events. For example, “FLU-LIKE SYMPTOMS” is annotated with the OAE term ‘influenza like illness AE’, which is supposed to be aligned with a MedDRA term “influenza like illness” (MedDRA ID: 10022004)^b^. This step is very important for normalizing event names for time trending analyses. For example, there could be multiple ways to describe “FLU-LIKE SYMPTOMS” such as “Influenza-like illness”, “flu-like illness”, etc. Only when these different expressions are annotated with the same ontology term, can a computer program know that they refer to the same meaning. The extracted relationships will also be represented in a computer-understandable way (in our case in the Rich Description Format or RDF, which is a standard data representation mechanism in Semantic Web), so that they can be easily queried too. Figure 4, for example, shows how we represent the relationship between “1ST ENGERIX-B DOSE” and its timestamp “3-29-90”.

### Visualization of time events

**Figure 5:**
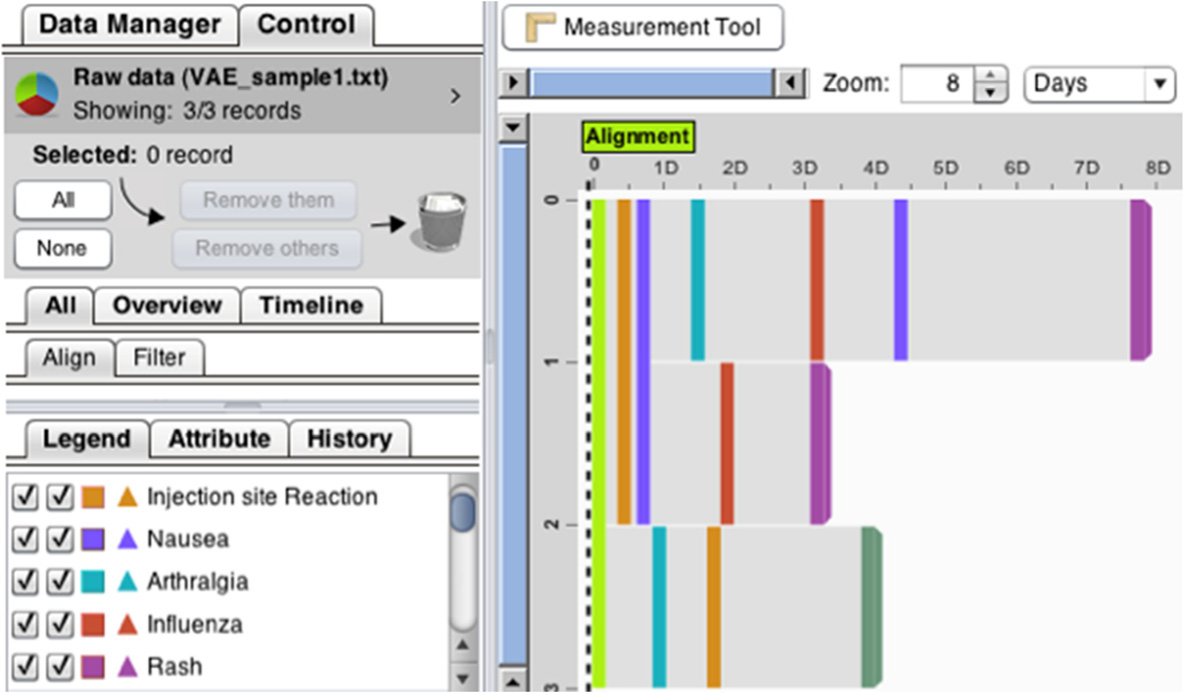
LifeFlow event sequence visualization environment with sample data.

With our ontology-based approach, we can represent both the structured data and the data extracted from the write-up narratives with respect to our ontologies to create an integrated data repository for further data analysis. Since the data is represented in a “machine-understandable” way, it is therefore possible for computer programs to query or infer temporal relationships between the events, or to sort events of interest on the timeline. We believe this is a very necessary step for improving the time trending analysis for vaccine adverse events. Figure 5, for example, shows the event sequences for three sample data files in an event sequence visualization environment called LifeFlow [21].

### Interpretation of time event analysis results

We randomly selected three VAERS reports for patient vaccination with HEP vaccine. Although analyzing three reports may not be statistically significant, we use these three samples to illustrate how we may interpret clinically significant or meaningful results from the data.

(i) Many symptoms are shared but may occur at different time points. For example, rash is shared by the first two patients, one at day 3, and the other at day 8. Influenza and arthralgia symptoms also are reported by the first two patients but at different days. The third patient had an injection site reaction on the second day instead of the first day (for the first two patients).

(ii) Frequency of a symptom may be different. Nausea occurred twice for the first patient. However, it occurred only once for the second patient and did not happen in the third patient.

(iii) Sequence of the adverse events may be different: The first patient had an injection site reaction then arthralgia. The two symptoms also showed up in the second patient in the opposite order.

Our approach provides a way to identify these differences, which can be used for further investigation.

## Conclusions and future work

In this paper, we introduce an ontology-based approach for representing time-related information from the VAERS repository. We believe that the ability to represent both the structured data and the data from write-up narratives in an integrated, unified, and “machine-understandable” way can enable research in vaccine safety analyses, causality assessments, and related retrospective studies. For example, the time-based analysis will improve the assessment of the AE causality. If we use a large amount of reports, a strong statistical correlation between vaccination and AEs can be identified with help of the temporal relations between AEs and vaccinations.

Based on the representation mechanisms defined in the ontology, we will implement tools for automatically extracting information such as event names, vaccine names, as well as temporal relationships from the VAERS system and semantically annotate the extracted information with respect to the three ontologies. Our Natural Language Processing team has developed a framework to extract temporal relation from discharge summaries [22]. We are currently working on linking this framework to the TIMER framework for semi-automatic information extraction. In addition, another future direction is to build a tool for statistical analysis on top of the integrated data integrated by representation with ontologies.

## Endnotes

^a^Some information about the TEO introduction is from the TEO web page: http://informatics.mayo.edu/CNTRO/index.php/TEO, which is maintained by Dr. Cui Tao

^b^Note that in Figure 2, “FLU-LIKE SYMPTOMS” in the write-up was annotated as MedDRA term “influenza” (in the symptom section). This is an inaccurate alignment done by the VAERS database as MedDRA has a more accurate term “influenza like illness” (MedDRA ID: 10022004).

## Competing interests

Dr. Poland is the chair of a Safety Evaluation Committee for an investigational vaccine trials being conducted by Merck Research Laboratories. Dr. Poland offers consultative advice on new vaccine development to Merck & Co., Inc., MedImmune LLC, Novavax, CSL Biotherapies, Avianax, Theraclone Sciences (formally Spaltudaq Corporation), Liquidia Technologies, Inc., Emergent BioSolutions, Dynavax, Novartis Vaccines and Therapeutics and PAXVAX, Inc. Dr. Poland also is a consultant for Ofstead and Associates and Sanofi Pasteur. This research has been reviewed by the Mayo Clinic Conflict of Interest Review Board and is being conducted in compliance with Mayo Clinic Conflict of Interest policies.

## Authors’ contributions

CT led the study design and analysis, and drafted the manuscript. YH contributed to the manuscript writing and the study design, especially to the VO and OAE sections. HY contributed to the use case annotation. GAP contributed to the manuscript preparation from a clinical point of view. CGC provided institutional support and manuscript editing. All authors read and approved the final manuscript.

## Authors’ information

The project was done when Hannah Yang was a visiting student intern in Mayo Clinic.
